# Rest as a Cause of Aggravation

**Published:** 1883-01

**Authors:** C. Von Bœnninghausen


### REST AS A CAUSE OF AGGRAVATION.

DR. C. VON BŒNNINGHAUSEN.

All that needs to be said concerning the influence of *rest* upon the aggravation of symptoms may be done with considerable brevity, for it is essentially the converse of what has been said regarding *motion.*

One variety of rest, however, demands a brief consideration, and all the more because, in the first place, it affords for many internal and external diseases a truly indispensable characteristic, and, in the second place, because it must astonish every experienced homœopathist to see, as in the more recent descriptions of clinical cases, that many practitioners appear to leave it altogether out of account. I refer to *rest* in a *recumbent position.*

I pass by *simple* lying, which is merely rest in contradistinction to motion, and also *lying in bed*, and propose to consider the *different recumbent positions*, which are points of greatest interest in this connection.

First under this comes aggravation from *lying outstretched* in contradistinction to *lying crooked* or in a *curved position.* For aggravation in the former posture, Cham., Colch., Platina, Puls., Rheum, Rhus, and Staph. are most likely to be appropriate. For aggravation in the latter, most frequently, Hyos., Lyc., Spong., Teucr., and Valer.

Aggravation from *lying with the head low* indicates other remedies again, among which are Ant. t., Arg., Ars., Caps., China, Colch., Hepar, Lach., Nitrum, Puls., and Spig. If in addition the horizontal position is most tolerable to the patient, then Apis, Arn., Bell., and Spongia may be added to the above.

But still more important than these are the recumbent positions upon the back and upon the *sides.*

If *lying upon the back* aggravates, this indicates especially Amm. m., Ars., Caust., Cham., China, Coloc., Cupr., Cycl., Iod., Nitr., Nux v., Phos., Plumb., Rhus, Sep., Sil., or Spig. When, on the contrary, this position affords relief, the most suitable remedy will generally be found among Acon., Anac., Bry., Calc., Carbo an., Kali c., Lyc., Merc., Puls., Seneg., Stann., Thuja.

Aggravation from *lying on the side* requires in *general* Acon., Anac., Bry., Calc., Carbo an., Kali c., Lyc., Phos., Puls., Stann., Sulph., and Thuja. But under this head there are two varieties which are of very great importance, viz.:

1. The aggravation may be produced by lying upon the *right* or upon the *left side;* 2. It may be produced by lying on the *painful side* or on the *side which is not the seat of pain.*

If we disregard these distinctions we grope about in the dark in treating many affections of the head, chest, abdomen, and limbs, and we fail to select the correct and successful remedy until we have long sought it and experimented in vain, whereas we might easily have found it at the outset had we paid attention to the distinctions above indicated.

The following remedies correspond to aggravation produced by lying on the *right side:* Amm. m., Borax, Caust., Mag. m., Nux v., and Spongia; by lying on the *left side,* Acon., Amm. c., Baryt., Bry., Colch., Ipec., Natr. c., Natr. m., Phos., Puls., Sep., Sil., Sulph., and Thuja. But when this condition comes into collision with the following one the preference is always to be given the *latter.*

The most important distinction, and one of which we may the most frequently avail ourselves in practice, is that between aggravation produced by *lying on the painful side* and that produced by *lying on the painless side.*

In the former case the chief remedies are: Acon., Amm. c., Ars., Baryt., Calad., Cycl., Dros., Graph., Hepar, Iod., Lach., Lyc., Magn. c., Magn. m., Mosch., Nitr. ac., Nux m., Nux v., Par., Phos., Ph. ac., Rheum, Ruta, Sabad., Selen., Sil., Staph., and Thuja.

On the other hand, aggravation when *lying on the painless side* occurs uniformly under the following remedies: Ambra, Arn., Bry., Calc., Cann., Caust., Cham., Coloc., Fluor. ac., Ign., Kali c., Puls., Rhus, Secale, Sep., Stann., and Viola tri.

All of these indications are so trustworthy and have been verified by such manifold experience that there are hardly any others that can equal them in rank, to say nothing of surpassing them. But the most valuable fact respecting them is this: that this characteristic is not confined to one or another symptom, but, like a red thread, it runs through all the morbid symptoms of a given remedy which are associated with any kind of pain whatever, or even with a sensation of discomfort, and hence it is available for both internal and external symptoms of the most varied character.

It is really a matter of surprise and wonder that an element so obvious and so very valuable for the appropriate selection of a remedy should have remained so entirely unheeded in many of the recent details of cases so carefully drawn up in other respects. Instead of these, the results of auscultation and percussion are recorded with the utmost exactness, notwithstanding the fact that the symptoms of our old and well-tried materia medica contain nothing whatever relating to these—then unknown—methods of observation, and that, consequently, they are almost worthless as items on which to base the choice of the remedy.

If the conscientious homœopathic physician is more intent on curing his patient as speedily and as safely as possible than on making a parade before him and astonishing him by a display of scientific accomplishments, he will, at least, first seek out in the case those—I had almost called them therapeutic-pathological—characteristic symptoms by which he may make sure his choice of the remedy, and not until he has done this will he seek to make available the general physiologico-pathological phenomena, for these, at least, can then do no mischief; and if he desires in a laudable manner to prepare the way for a future useful application of the stethoscope and the fleximeter, let him seek to bring the results of their employment into relation with the above-mentioned old and verified symptoms in such wise that both united may be employed in making the cure even more certain and precise than before.

He, however, who does not go to work in this fashion, but, in contradiction to section 153 of the *Organon*, pursues his way over the barren waste of a pathology without characteristic, should as little expect to be recognized as a true homœopathic physician as those others who, in opposition to section 245 *et sequa* of the *Organon*, by their, to say the least, *unnecessary* administration of massive doses, give our adversaries occasion to declare that the distinction between allopathic and homœopathic remedies exist, and to deduce from this the futility of the claim on the part of physicians to the liberty of dispensing their own remedies, since, in fact, the necessity for this liberty is denied to exist.

If any one, whoever he may be, has the audacity to proclaim to the world that Hahnemann himself, toward the end of his life, returned to the use of massive doses and only maintained an outside semblance of adherence to his potentiation theory, from unworthy motives, such a man is nothing but a common slanderer, unworthy of respect and credit at the hands of any honorable man, whether allopath or homœopath, and he will be nailed to the pillory as a malignant liar by the publication, which we may very soon expect, of original matter from our great master's own journals.*

* Published in *Am. Hom. Review*, by Dr. Dunham.

